# Development and validation of a Spanish-language questionnaire to assess mothers’ knowledge and practices regarding their children’s oral health

**DOI:** 10.21142/2523-2754-1403-2026-299

**Published:** 2026-07-10

**Authors:** Martha J. Arias-Mendoza, Andrés A. Agudelo-Suárez, Emilia M. Ochoa-Acosta, Ethman A. Torres-Murillo, Sonia C. Concha-Sánchez

**Affiliations:** 1 Faculty of Dentistry, Universidad Santo Tomás. Santander, Colombia. ethman.torres@ustabuca.edu.co Universidad Santo Tomás Faculty of Dentistry Universidad Santo Tomás Santander Colombia ethman.torres@ustabuca.edu.co; 2 Faculty of Dentistry, Universidad Cooperativa de Colombia. Medellín, Colombia. alonso.agudelo@udea.edu.co, emilia.ochoa@campusucc.edu.co Universidad Cooperativa de Colombia Faculty of Dentistry Universidad Cooperativa de Colombia Medellín Colombia alonso.agudelo@udea.edu.co emilia.ochoa@campusucc.edu.co; 3 Public Health Department, Universidad de Santander. Bucaramanga, Colombia. sococosa@yahoo.com Universidad de Santander Public Health Department Universidad de Santander Bucaramanga Colombia sococosa@yahoo.com

**Keywords:** Health Knowledge, Attitudes, Practice, Oral Health, Health Education, Health Promotion, Validation Study, conocimientos, actitudes y práctica en salud, salud bucal, educación en salud, promoción de la salud, estudio de validación

## Abstract

**Objective:**

This study aimed to design and validate a Spanish-language questionnaire to assess mothers’ knowledge and practices regarding their children’s oral health.

**Material and methods:**

An exploratory study with a descriptive scope was conducted using both quantitative and qualitative data collection techniques and instruments, following these phases: 1) Review of relevant scientific literature; 2) Identification of core items and development of the preliminary instrument; 3) Preliminary pilot testing with an expert panel; 4) Semantic testing of the questionnaire in the target population; 5) Assessment of the instrument’s reliability and reproducibility; 6) Validation of the instrument’s appearance and content through two rounds with subject matter experts, including the calculation of various validity indicators: Content Validity Index (CVI), Content Validity Ratio (CVR), and Content Validity Coefficient (CVC).

**Results:**

An assessment instrument was developed comprising 31 items that capture information across three dimensions: 1) psychosocial (attitudinal and relational); 2) knowledge; and 3) practices. All dimensions addressed aspects related to breastfeeding and nutrition, hygiene, and oral health within the mother-child dyad. Expert validation rounds produced CVI, CVR, and CVC values calculated for each item (I), dimension (S), and for the overall instrument (S). All scores exceeded the established thresholds (CVI ≥ 0.78/0.90; CVR ≥ 0.37/0.70; and CVC ≥ 0.90), indicating adequate content validity. Minor wording and structural adjustments were made to some questions based on expert feedback.

**Conclusion:**

This study resulted in the development of a Spanish-language assessment instrument with adequate content and face validity, which is useful for designing educational strategies targeting the mother-child dyad to promote proper oral health.

## RESUMEN

**Objetivo:**

Diseñar y validar un cuestionario en español para evaluar conocimientos y prácticas de las madres sobre la salud bucal de sus hijos.

**Materiales y métodos:**

Estudio exploratorio con alcance descriptivo, con técnicas e instrumentos de recolección cuantitativos y cualitativos, y con las siguientes fases: 1) Revisión de la literatura científica relevante; 2) Selección de preguntas clave y construcción del instrumento inicial; 3) Pilotaje preliminar con un panel de expertos; 4) Evaluación semántica del cuestionario en la población objetivo; 5) evaluación de la confiabilidad y reproducibilidad del instrumento; 6) Validación de la apariencia y el contenido del instrumento mediante dos rondas con expertos en el tema, incluyendo el cálculo de diversos indicadores de validez: Índice de Validez de Contenido (CVI), Razón de Validez de Contenido (CVR) y Coeficiente de Validez de Contenido (CVC).

**Resultados:**

Se desarrolló un instrumento de evaluación compuesto por 31 ítems que recoge información en tres dimensiones: 1) psicosocial (actitudinal y relacional); 2) conocimientos; y 3) prácticas. Todas las dimensiones abordaron aspectos relacionados con la lactancia materna y la alimentación, higiene y salud oral en el binomio madre-hijo. Las rondas de validación por expertos produjeron valores de CVI, CVR y CVC calculados para cada ítem (I), cada dimensión (S) y para el instrumento global (S). Todos los puntajes superaron los umbrales establecidos (CVI ≥ 0,78/0,90; CVR ≥ 0,37/0,70; y CVC ≥ 0,90), lo que indica una adecuada validez de contenido. Con base en la retroalimentación de los expertos, se realizaron ajustes menores en la redacción y estructura de algunas preguntas.

**Conclusión:**

se desarrolló un instrumento en español adecuado en contenido y apariencia, útil para promover la salud bucal en los niños a través de estrategias educativas en las madres.

## INTRODUCTION

Health promotion primarily refers to a positive concept that encompasses a political, social, and global process involving actions aimed at strengthening individual and collective skills and capacities to improve health conditions ^1^. By encompassing notions such as well-being, quality of life, and human development, it becomes essential to reinforce knowledge, attitudes, and health practices as fundamental contributions to the design and implementation of effective strategies that address the specific needs of various social groups ^1^.

Knowledge, attitudes, and practices (KAP) surveys are widely employed in public health research across diverse social contexts and in relation to specific health behaviors or conditions. ^2^^,^^3^ In oral health, this is no exception. For instance, a simple search using various descriptors in the PubMed database reveals narrative, scoping, and systematic reviews on general topics and specific conditions such as dental caries, periodontal disease, and oral cancer, which consider different population groups. ^4^^-^^10^ It is noteworthy that qualitative studies are mentioned, involving people’s perceptions and opinions in their everyday lives. ^11^^-^^13^

One of the most vulnerable social groups is pregnant women. This stage of adaptation may involve biological and social conditions that increase their susceptibility to adverse oral health conditions. ^14^ In other cases, they may face barriers to accessing dental services. ^15^ This situation can be even worse in socially disadvantaged groups, such as minority ethnic groups ^8^, migrants, or those with low socioeconomic status or education levels. ^16^^,^^17^ Nevertheless, this group can be highly receptive to the implementation of health promotion strategies, as they are part of mother-child initiatives that encourage healthy behaviors and can contribute to better oral health in children. ^18^ Regarding pregnant and maternal women, it is noteworthy that a considerable number of studies focus on assessing their own oral health KAP ^19^^-^^22^, but less frequently investigate the knowledge and practices they should have concerning their current and future children’s oral health.

Health promotion and prevention programs must begin with an understanding of the specific social and cultural conditions of each country or territory. This entails the development of data collection instruments that address specific needs-in this case, oral health-that can be effectively analyzed and consolidated. From this perspective, a larger project was designed to assess the effects of an educational intervention on mothers’ knowledge and practices concerning their children’s oral hygiene and how these changes may improve oral health outcomes in children under two years old. The project combines quantitative and qualitative approaches and promotes women’s active engagement in interdisciplinary health programs.

To the best of our knowledge, there are few validated instruments in Spanish for pregnant and maternal women related to this research topic. Accordingly, this study aims to design and validate an instrument to assess maternal knowledge and practices regarding their children’s oral health.

## MATERIAL AND METHODS

**Figure 1:**
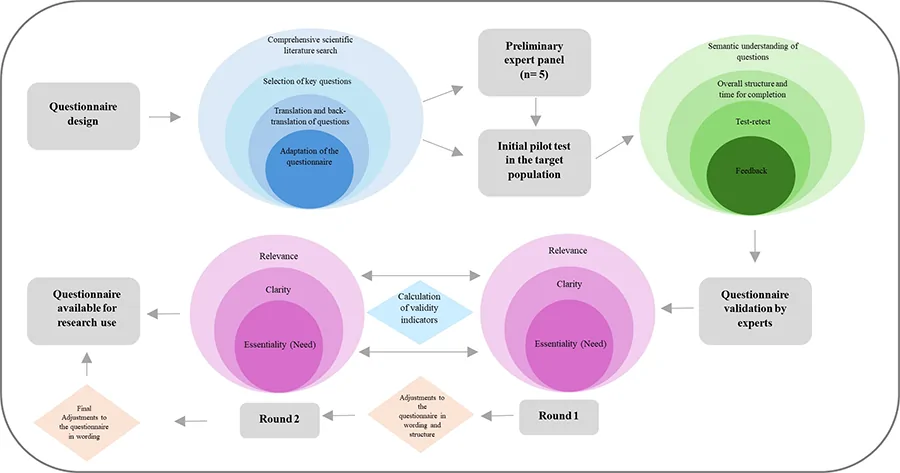
Flowchart illustrating the phases of the questionnaire design and validation

This exploratory study employed both quantitative and qualitative data collection techniques and instruments. Its implementation follows the process recommended in the Association for Medical Education in Europe (AMEE) Guide No. 87 ^23^, and some recommendations focused on the construction and validation of measurement instruments in the health field ^24^. Some adaptations were made to accommodate the overall scope and limitations of the research process. Figure 1 provides a summary of the study’s steps, with a detailed description below: 

### Phase 1: Search for related scientific literature

A literature search was conducted for the period 2012-2023 to identify instruments focused on the mother-child dyad, specifically assessing mothers’ knowledge and practices related to their children’s oral health (particularly in children under five years of age). The search included studies published in English, Portuguese, or French that provided the questionnaire or detailed the questions. Complex keyword equations were not applied, as the study did not aim to conduct a systematic review. This review was led by the first author (M.J.A.M.). 

The search yielded 16 articles. ^25^^-^^40^ After an initial review, the questions from the data collection instruments in 14 of these studies were examined for relevance to the study objectives, while two were used as theoretical references. Ultimately, 13 questions from four studies were selected for analysis and discussion. ^27^^,^^28^^,^^37^^,^^39^

### Phase 2: Identification of core items and development of the preliminary instrument

Some items were originally written in a second language, so their translation was performed by a qualified expert. A document containing 13 questions in English was submitted for translation, after which the Spanish version was back translated into English by a native speaker to ensure linguistic and conceptual accuracy. The manuscript authors then conducted an initial review of the translated material.

An advisory panel composed of five experts was established to guide the instrument development process. The panel included two pediatric dentistry specialists (one male and one female), two dentists with expertise in epidemiology and public health (one male and one female, both with PhDs), and one dentist with a PhD in education (female).

Under the coordination of the first author, five consensus meetings were held to review and refine the questionnaire items. During these sessions, the initial questions identified in the literature were critically evaluated, and new items were proposed by consensus. This process resulted in a total of 31 items distributed across three dimensions: 1) psychosocial, attitudinal, and relational aspects (nine items): These refer to emotions related to breastfeeding and the children’s oral health status; 2) knowledge: These questions assess how much pregnant and maternal women know about oral health and/or specific pathologies, such as dental caries; and 3) practices: This dimension includes items designed to assess both appropriate and inappropriate practices commonly observed in relation to oral hygiene and oral health during early childhood. 

Whenever possible, the items were formatted using a Likert-type scale. Response options reflected levels of agreement, satisfaction, perceived importance, or the frequency of specific events or situations, all pertaining to pregnant or maternal women’s opinions and perceptions regarding the oral health of their children (both current and future). In addition to these three dimensions, nine questions addressing basic sociodemographic variables were included. These items can be adapted as needed to suit specific research contexts.

### Phase 3: Preliminary pilot testing with expert panel

After the instrument was developed, organized, and approved by the advisory team, an invitation was extended to five pediatric dentists with expertise in the questionnaire topic (academic, research, and clinical). These experts had previously participated in a focus group for the qualitative phase of the general project mentioned in the background of this manuscript.

Experts were invited via email to evaluate the instrument using a color-coded assessment system (“traffic light” system), where green indicated clarity and relevance, yellow suggested that the item was acceptable but could be improved, and red indicated that it was unnecessary or not pertinent. Overall, 89.5% of the items were rated green, while the remaining 10.5%, rated yellow or red, were deemed acceptable after minor revisions to wording or terminology.

Additionally, qualitative feedback was obtained. Participants proposed several refinements, such as expanding items on infant oral hygiene, including questions on breastfeeding status, and addressing the fluoride concentration in toothpaste. They also emphasized the importance of assessing family practices and the influential role of grandmothers, in transmitting knowledge and caregiving traditions that mothers may choose to maintain. These insights highlight opportunities for future qualitative and mixed-method studies focusing on oral health care among parents and other significant adults. Overall, experts deemed the questionnaire appropriate.

Following the review by the research team, we obtained the initial version of this 31-item questionnaire, as shown in Table 1.

**Table 1:** List of dimensions and items of the initial core questionnaire

Dimension	Item	Question	Response options
Psychosocial	D1	Breastfeeding increases the mother-child bond.	Strongly disagree / Partially disagree / Neither disagree nor agree / Partially agree / Strongly agree
	D2	Maintaining a child’s oral health is a duty of parents.	Strongly disagree / Partially disagree / Neither disagree nor agree / Partially agree / Strongly agree
	D3	When feeding my baby with a bottle, I will feel:	Totally dissatisfied / Dissatisfied / Somewhat satisfied / Satisfied / Totally satisfied
	D4	I believe that as a mother, when breastfeeding, I will feel:	Totally dissatisfied / Dissatisfied / Somewhat satisfied / Satisfied / Totally satisfied
	D5	Acquiring knowledge about oral care and hygiene practices for my baby is:	Not useful at all / Not very useful / Somewhat useful / Very useful / Extremely useful
	D6	Learning about how to feed my baby during the first year of life is:	Not useful at all / Not very useful / Somewhat useful / Very useful / Extremely useful
	D7	I believe that breastfeeding my baby is:	Not important / Slightly important / Somewhat important / Important / Very important
Knowledge	K1	Current formula milks are as nutritious as breast milk.	Strongly disagree / Partially disagree / Neither disagree nor agree / Partially agree / Strongly agree
	K2	Breast milk has greater benefits than formula milk.	Strongly disagree / Partially disagree / Neither disagree nor agree / Partially agree / Strongly agree
	K3	The mother naturally and instinctively learns how to breastfeed her baby.	Strongly disagree / Partially disagree / Neither disagree nor agree / Partially agree / Strongly agree
	K4	Babies fed with breast milk are healthier than those fed with formula milk.	Strongly disagree / Partially disagree / Neither disagree nor agree / Partially agree / Strongly agree
	K5	Breastfeeding becomes difficult when the mother works outside the home.	Strongly disagree / Partially disagree / Neither disagree nor agree / Partially agree / Strongly agree
	K6	Feeding my child at night with a bottle of formula milk or a drink containing sugar affects his/her teeth.	Strongly disagree / Partially disagree / Neither disagree nor agree / Partially agree / Strongly agree
	K7	A child’s oral health is related to general health.	Strongly disagree / Partially disagree / Neither disagree nor agree / Partially agree / Strongly agree
	K8	Stains on the surfaces of teeth are the first signs of dental caries.	Strongly disagree / Partially disagree / Neither disagree nor agree / Partially agree / Strongly agree
	K9	Dental caries is caused by bacteria transmitted when feeding utensils (e.g., spoons) are shared.	Strongly disagree / Partially disagree / Neither disagree nor agree / Partially agree / Strongly agree
	K10	Fluoride toothpaste will help prevent dental caries in my child.	Strongly disagree / Partially disagree / Neither disagree nor agree / Partially agree / Strongly agree
	K11	The mother’s diet during pregnancy affects the development of the baby’s teeth.	Strongly disagree / Partially disagree / Neither disagree nor agree / Partially agree / Strongly agree
	K12	Feeding my child with breast milk will help with better growth of the face and teeth.	Strongly disagree / Partially disagree / Neither disagree nor agree / Partially agree / Strongly agree
	K13	Habits (lip sucking, finger sucking, cloth sucking, and pacifier use) affect the development of teeth and facial bones.	Strongly disagree / Partially disagree / Neither disagree nor agree / Partially agree / Strongly agree
Practices	P1	If you work outside the home, how will you feed the baby?	Formula milk / Breastfeeding when with the baby and formula when not with him/her / Expressed breast milk
	P2	About primary and permanent teeth, I think that:	Primary teeth are not important at all / Primary teeth are slightly important / Primary and permanent teeth are equally important
	P3	Until what age will you feed your baby with breast milk?	Three months / Six months / Twelve months / Two years / Until the child wants
	P4	Who would carry out the baby’s oral care practices?	Yourself / A family member / An external caregiver / Other
	P5	How will you feed your child up to six months of age?	Only breast milk / Mainly formula milk / Breast milk and formula milk / Other
	P6	How will you feed your child from six months to one year of age?	Only breast milk / Breast milk and formula milk / Breast milk plus complementary feeding / Only complementary feeding
	P7	At what age will you take your child to the first dental visit?	At birth / Between six months and one year of age / After six years of age / When he/she feels pain
	P8	When do you think cleaning the baby’s mouth should begin?	As soon as the first tooth erupts / After the eruption of all primary teeth / The gums should be cleaned regularly after birth / I don’t know
	P9	How often should the baby’s mouth be cleaned?	Once a day / Regularly after each feeding / Cleaning is not required before teeth erupt / I don’t know
	P10	How will you clean your baby’s mouth before the teeth erupt?	With water / With wet gauze / With a toothbrush / With toothpaste / It should not be done
	P11	If you work outside the home, how will you feed the baby?	Formula milk / Breastfeeding when with the baby and formula when not with him/her / Expressed breast milk

### Phase 4: Semantic test of the questionnaire in the target population

This pilot test was conducted with eight pregnant women in the waiting rooms of a local hospital in Piedecuesta, located in the department of Santander, in eastern Colombia near the Venezuelan border. Piedecuesta is a municipality situated 17 kilometers from Bucaramanga, the capital of Santander, with a population of approximately 186,000 inhabitants.

This phase aimed to analyze technical aspects related to the clarity, syntax, and semantics of the questions. Clarity referred to whether participants found the questions easy to understand or confusing. Syntax and semantics assessed whether the wording of the questions was correct and whether the terms used were clear and appropriate. Each question was followed by an evaluation scale ranging from 1 to 5 for each criterion, with 5 representing the highest score and 1 the lowest. It was observed that some mothers did not recognize certain terms, such as breastfeeding, fluoridated toothpaste, and formula milk. The mean clarity score among participants was 4.8, while the mean syntax and semantics score was 4.7.

### Phase 5: Reliability and reproducibility of the instrument

A previous cross-sectional study was conducted in two public healthcare institutions (the Clínica Girón and the Local Hospital of Piedecuesta, Department of Santander, Colombia). Participants were recruited through convenience sampling of pregnant women enrolled in maternity and paternity preparation programs. ^41^ The internal consistency of the 31-item questionnaire was evaluated in a sample of 269 women based on a single administration. The analysis yielded a Cronbach’s alpha of 0.774 and a McDonald’s omega of 0.758, demonstrating an acceptable level of internal reliability. The reproducibility of the instrument was assessed through a test-retest procedure with a sample of 30 women. The instrument demonstrated good overall reproducibility, with an intraclass correlation coefficient (ICC) of 0.702 (95% CI: 0.374-0.858). 

### Phase 6: Validation of the instrument’s appearance and content through subject matter experts

Two rounds of expert validation were conducted using an online survey administered via the Google Forms platform, which facilitated efficient and timely data collection while minimizing potential response-entry errors. The research team recruited 25 experts, purposefully selected based on the study objectives. A summary of the participating experts’ profiles is provided in Table 2. 

**Table 2:** Characteristics of experts participating in the validation phase (two rounds, n = 25)

ID	Academic profile	Sex	Age	Origin Country
APL01	Dentist. MSc in Epidemiology. PhD in Dental Sciences	Female	48	Colombia
SUA02	Dentist. MSc in Biomedical Engineering	Male	35	Colombia
KD03	Dentist. Specialist in Public Oral Health. MSc in Project Management	Male	33	Peru
GSG04	Psychologist. MSc in International Migration Studies	Female	40	Peru
AMP05	Dentist. Specialist in Statistics. MSc in Dental Sciences	Male	34	Colombia
AVA06	Dentist. PhD in Dental Sciences	Female	55	Ecuador
AAB07	Dentist. Pediatric Dentist	Female	35	Colombia
PHH08	Dentist. Pediatric Dentist	Female	45	Colombia
GMM09	Dentist. MSc in Administration. PhD in Public Health	Male	42	Colombia
JSB10	Dentist. Pediatric Dentist. PhD in Dental Sciences	Female	41	Colombia
CSC11	Dentist. Pediatric Dentist	Female	44	Colombia
ODD12	Dentist. MSc in Telehealth	Female	40	Colombia
RRM13	Dentist. Pediatric Dentist	Female	65	Peru
JVA14	Sociologist. MSc in Public Health	Male	62	Colombia
CFR15	Dentist. Specialist in Health Education and Communication	Female	47	Colombia
DGA16	Dentist. Specialist in Quality and Patient Safety. MSc in Dental Sciences	Male	48	Colombia
GBF17	Dentist. Pediatric Dentist. MSc in Dental Sciences. PhD (c) in Health Sciences	Female	30	Colombia
CMD18	Dentist. Specialist in Epidemiology. MSc in University Management	Female	71	Colombia
DRT19	Medical Doctor, Pediatrician	Female	71	Colombia
BGF20	Dentist. Specialist in Pediatric Stomatology and Maxillary Orthopedics	Male	35	Colombia
GTR21	Nurse. Experience in Maternal Health Programs	Female	25	Colombia
DBO22	Dentist. MSc in Dental Sciences. PhD In Health Policy (student)	Male	31	Colombia
MPB23	Dentist. Pediatric Stomatologist	Female	52	Colombia
DPP24	Dentist. Specialist in Pediatric Dentistry and Orthopedics	Female	31	Colombia
FTR25	Dentist. Specialist in Health Care Institution Management	Female	40	Colombia

The 31 items of the questionnaire and their corresponding response options were evaluated based on three criteria, each assessed using Likert-type scales: 1) Relevance: 1 = not relevant, 2 = slightly relevant, 3 = relevant, 4 = highly relevant; 2) Clarity: 1 = not clear, 2 = somewhat clear, 3 = clear, 4 = very clear; 3) Essentiality (Need): 1 = not necessary, 2 = useful but not essential, 3 = necessary. Basic sociodemographic variables (age, sex, undergraduate and postgraduate degrees, years of professional experience, and workplace) were included to characterize the participants. Additionally, open-ended questions were incorporated to solicit suggestions and comments regarding the 31 items and the three dimensions of the core questionnaire.

The experts’ responses concerning the criteria of relevance, clarity, and essentiality (need) were used to calculate several indicators that collectively help determine whether the items and dimensions are valid, require modifications, or should be eliminated. All these indicators are thoroughly explained in Table 3. Briefly, the following were utilized:

- Content Validity Index (CVI): This index assesses the degree of agreement among experts and ranges from 0 to 1 _^(42)^_ . It was calculated for each item, dimension, and overall. _^(43)^_
- Content Validity Ratio (CVR): This index quantifies the degree of expert agreement on the essentiality of each item within an instrument that measures a specific construct. _^(44)^_ Proposed by Lawshe (1975), it has undergone some subsequent modifications. _^(45)^_
- Content Validity Coefficient (CVC): this indicator was proposed by Hernández-Nieto. _^(46)^_ In this case, a macro developed in Microsoft Excel by Maldonado-Suárez and Santoyo-Téllez was used _^(47)^_ , which can be downloaded from the following link: https://zenodo.org/doi/10.5281/zenodo.12535410.

**Table 3:** Explanation of the indicators used in the expert validation

Indicator		Formula	Explanation	Interpretation (for 29 experts)	
Item-level Content Validity Index	I-CVI	I-CVI = *ne* *N*	ne: Number of experts who rated the item as 3 or 4 (relevance, clarity, essentiality) N: Total number of experts who evaluated the item	≥ 0.78	Acceptable
Scale-level Content Validity Index	S-CVI/Ave	S-CVI/Ave = *∑I-CVI* *N*	∑ I-CVI: Sum of all I-CVI values N: Total number of items in the instrument	≥ 0.90	Excellent content validity
Scale-level Content Validity Index, Universal Agreement	S-CVI/UA	S-CVI/UA = *NUA* *N*	NUA: Number of items that achieved universal agreement among experts N: Total number of items in the instrument.	The research team opted to use the S-CVI/Ave, given that the S-CVI/UA is overly sensitive when a large number of experts participate.	
Item-level Content Validity Ratio	I-CVR	I-CVR = *ne-N⁄2* *N⁄2*	ne: Number of experts rating the item as essential N: Total number of experts who evaluated the item	≥ 0.37	Acceptable
Scale-level Content Validity Ratio	S-CVR/Ave	S-CVR/Ave = *∑I-CVR* *N*	∑ I-CVR: Sum of all I-CVR values N: Total number of items in the instrument	≥ 0.70	Acceptable
Item-level Content Validity Coefficient	I- CVC	I- CVC = *∑×i/j* - Pei *Vmax* *Pe =* (1 ^*j*^ *j* )	Xi: mean score of the item given by the experts j: Total number of experts who evaluated the item Pe: error term for the item	≥ 0.90	Excellent content validity
Scale-level Content Validity Coefficient	S-CVC/Ave	S-CVC/Ave = ∑I-CVC N	∑ I-CVC: Sum of all I-CVC values N: Total number of items in the instrument	≥ 0.90	Excellent content validity

### Ethical aspects

This project employed both qualitative and quantitative tools; therefore, the study protocol was approved by the Committee on Ethics, Bioethics, and Scientific Integrity (CEBIC, acronym in Spanish) of Santo Tomás University, in accordance with Act No. 28 (December 13, 2022) in Bucaramanga, Colombia. Informed consent was ensured throughout the research process, provided in written form, online via Google Forms, or by email. This study complies with national and international regulations governing research involving human participants, including Resolution 8430 of the Ministry of Health of Colombia, the Declaration of Helsinki, and the guidelines of the Council for International Organizations of Medical Sciences (CIOMS). ^48^^-^^50^

## RESULTS

The following results were derived from the evaluation phases conducted by subject-matter experts. In sociodemographic terms, the panel consisted of eight men and 17 women, with a mean age of 44 ± 13 years and an average of 18.6 ± 10 years of professional and academic experience. Twenty-three experts were dentists, while the panel also included a sociologist, a nurse, and a pediatrician. Twenty-one experts were affiliated with academic institutions, and seven were associated with public or private healthcare institutions.

### First round of expert evaluation of the core questionnaire

Table 4 presents the specific results of the calculated indicators for the overall questionnaire, by dimension, and by item. During this phase, the subject-matter experts provided an overall evaluation of the instrument, with scores ranging from 0.79 to 0.96, depending on the type of indicator and the factor assessed. The lowest value was observed for the essentiality factor, as reflected in the CVR (0.79). When analyzed by dimensions, the practices component obtained the lowest values for clarity and CVR (0.67); however, when considering essentiality and the CVC, the score reached 0.97. At the item level, the lowest indicator and factor values were found for D3 and D4 in the psychosocial dimension, K1, K3, K6, K8, and K9 in the knowledge dimension, and P11 in the practices dimension.

**Table 4:** Indicators of face and content validity of the questionnaire and its components obtained in the first round of expert evaluation

Dimension	Question	Relevance			Clarity			Essentiality (Need)			Review and decision of the research team (regarding the item, the dimension, and the instrument)
		I-CVI	I- CVR	I- CVC	I-CVI	CVR	I- CVR	I-CVI	I- CVR	I- CVC	
Psychosocial	D1	1.00	1.00	0.98	0.92	0.84	0.92	1.00	1.00	1.00	Keep
	D2	1.00	1.00	0.99	1.00	1.00	0.97	1.00	1.00	1.00	Keep
	D3	0.68	0.36	0.74	0.80	0.60	0.79	0.60	0.20	0.83	Modify
	D4	0.88	0.76	0.89	0.88	0.76	0.90	0.84	0.68	0.93	Modify
	D5	1.00	1.00	0.98	0.96	0.92	0.96	1.00	1.00	1.00	Keep
	D6	1.00	1.00	0.99	1.00	1.00	1.00	1.00	1.00	1.00	Keep
	D7	0.96	0.92	0.95	0.96	0.92	0.94	0.96	0.92	0.99	Keep
	S-CVI/Ave										Adequate, but some items may need revision
	S-CVR/Ave										
	S-CVC/Ave	0.93	0.86	0.93	0.93	0.86	0.93	0.91	0.83	0.96	
	S-CVI-UA	0.57	---	---	0.29	---	---	0.57	---	---	
Knowledge	K1	0.84	0.68	0.85	0.88	0.76	0.89	0.80	0.60	0.91	Modify
	K2	1.00	1.00	1.00	1.00	1.00	0.99	1.00	1.00	1.00	Keep
	K3	0.92	0.84	0.86	0.80	0.60	0.84	0.76	0.52	0.91	Modify
	K4	0.96	0.92	0.93	0.92	0.84	0.92	0.92	0.84	0.96	Keep
	K5	1.00	1.00	0.99	1.00	1.00	0.99	1.00	1.00	1.00	Keep
	K6	0.92	0.84	0.88	0.88	0.76	0.88	0.84	0.68	0.93	Modify
	K7	0.96	0.92	0.97	0.96	0.92	0.97	0.96	0.92	0.97	Keep
	K8	0.96	0.92	0.94	0.84	0.68	0.86	0.92	0.84	0.97	Modify
	K9	0.88	0.76	0.86	0.88	0.76	0.87	0.76	0.52	0.91	Modify
	K10	1.00	1.00	0.98	0.92	0.84	0.91	0.96	0.92	0.99	Keep
	K11	1.00	1.00	0.99	1.00	1.00	0.99	0.96	0.92	0.99	Keep
	K12	1.00	1.00	0.97	1.00	1.00	0.97	0.96	0.92	0.99	Keep
	K13	0.96	0.92	0.97	0.92	0.84	0.95	0.96	0.92	0.97	Keep
	S-CVI/Ave										Adequate, but some items may need revision
	S-CVR/Ave										
	s-CVC/Ave	0.95	0.91	0.94	0.92	0.85	0.93	0.91	0.82	0.96	
	S-CVI-UA	0.38	---	---	0.31	---	---	0.15	---	---	
Practices	P1	1.00	1.00	0.94	0.80	0.60	0.83	0.92	0.84	0.97	Modify
	P2	0.92	0.84	0.92	0.80	0.60	0.83	0.88	0.76	0.95	Modify
	P3	0.96	0.92	0.96	0.84	0.68	0.88	0.96	0.92	0.97	Keep
	P4	0.96	0.92	0.95	0.80	0.60	0.86	0.92	0.84	0.97	Keep
	P5	0.96	0.92	0.96	0.84	0.68	0.89	0.92	0.84	0.96	Keep
	P6	0.96	0.92	0.93	0.80	0.60	0.86	0.88	0.76	0.95	Keep
	P7	0.96	0.92	0.91	0.84	0.68	0.87	0.92	0.84	0.96	Keep
	P8	0.96	0.92	0.97	0.92	0.84	0.95	0.96	0.92	0.99	Keep
	P9	1.00	1.00	0.98	0.92	0.84	0.94	1.00	1.00	1.00	Keep
	P10	0.96	0.92	0.96	0.84	0.68	0.90	0.92	0.84	0.97	Keep
	P11	0.88	0.76	0.92	0.76	0.52	0.82	0.88	0.76	0.93	Modify
	S-CVI/Ave										Adequate, but some items may need revision
	S-CVR/Ave										
	S-CVC/Ave	0.96	0.91	0.95	0.83	0.67	0.88	0.92	0.85	0.97	
	S-CVI-UA	0.18	---	---	0.00	---	---	0.09	---	---	
S-CVI/Ave (Overall)											Adequate, but some items and dimensions may need revision
S-CVR/Ave (Overall)											
T- CVC (Overall)		0.95	0.89	0.94	0.91	0.83	0.91	0.89	0.79	0.96	
S-CVI-UA (Overall)		0.35	---	---	0.19	---	---	0.22	---	---	

Based on the analysis of these indicators and factors, the research team concluded that the dimensions, the overall questionnaire, and the individual items were suitable for the study objectives. Nevertheless, items with lower scores should be revised to enhance clarity and relevance, consistent with the project’s conceptual framework. Additionally, the experts provided qualitative feedback on both the dimensions and specific items, which informed subsequent modifications that were later re-evaluated and approved by the research team.

### Second round of expert evaluation of the core questionnaire

Certain modifications were made to the instrument regarding the wording of several questions. Additionally, one question was moved from the knowledge dimension to the psychosocial dimension, and another was transferred from the practices dimension to the knowledge dimension. The new questions correspond to items D8 and K13. A detailed comparison between the original and revised versions of the questionnaire, including the specific additions or changes made to question wording and response options, is provided in the Table 5.

**Table 5:** Comparative analysis for the List of questions and response options (initial and revised versions)

Dimension	Item	Questions			
		Original		Modified	
		Question	Response Options	Question	Response Options
Psychosocial	D1	Breastfeeding increases the mother-child bond.	Strongly disagree / Partially disagree / Neither disagree nor agree / Partially agree / Strongly agree	The original wording of the question and its response options were retained.	
	D2	Maintaining a child’s oral health is a duty of parents.	Strongly disagree / Partially disagree / Neither disagree nor agree / Partially agree / Strongly agree	The original wording of the question and its response options were retained	
	D3	When feeding my baby with a bottle, I will feel:	Totally dissatisfied / Dissatisfied / Somewhat satisfied / Satisfied / Totally satisfied	If it were necessary to bottle-feed your baby, how would you feel?	Totally dissatisfied / Dissatisfied / Neutral / Satisfied/ Totally satisfied
	D4	I believe that as a mother, when breastfeeding, I will feel:	Totally dissatisfied / Dissatisfied / Somewhat satisfied / Satisfied / Totally satisfied	Breastfeeding your baby makes you feel:	Totally dissatisfied / Dissatisfied / Neutral / Satisfied/ Totally satisfied
	D5	Acquiring knowledge about oral care and hygiene practices for my baby is:	Not useful at all / Not very useful / Somewhat useful / Very useful / Extremely useful	The original wording of the question was retained.	Not useful at all / Not very useful / Neutral / Somewhat useful / Very useful
	D6	Learning about how to feed my baby during the first year of life is:	Not useful at all / Not very useful / Somewhat useful / Very useful / Extremely useful	The original wording of the question was retained.	Not useful at all / Not very useful / Neutral / Somewhat useful / Very useful
	D7	I believe that breastfeeding my baby is:	Not important / Slightly important / Somewhat important / Important / Very important	The original wording of the question was retained	Not important / Slightly important / Neutral / Important / Very important
	D8	Breastfeeding becomes difficult when the mother works outside the home.	Strongly disagree / Partially disagree / Neither disagree nor agree / Partially agree / Strongly agree	Do you find it difficult to continue breastfeeding when you work outside the home?	The response options were retained; the item was initially in the Knowledge dimension and was relocated to this dimension.
Knowledge	K1	Current formula milks are as nutritious as breast milk.	Strongly disagree / Partially disagree / Neither disagree nor agree / Partially agree / Strongly agree	Do you think that formula milk is as nutritious as breast milk?	The response options were retained
	K2	Breast milk has greater benefits than formula milk.	Strongly disagree / Partially disagree / Neither disagree nor agree / Partially agree / Strongly agree	The original wording of the question and its response options were retained.	
	K3	The mother naturally and instinctively learns how to breastfeed her baby.	Strongly disagree / Partially disagree / Neither disagree nor agree / Partially agree / Strongly agree	Do you think that breastfeeding is learned naturally or instinctively?	The response options were retained
	K4	Babies fed with breast milk are healthier than those fed with formula milk.	Strongly disagree / Partially disagree / Neither disagree nor agree / Partially agree / Strongly agree	The original wording of the question and its response options were retained.	
	K5	Feeding my child at night with a bottle of formula milk or a drink containing sugar affects his/her teeth.	Strongly disagree / Partially disagree / Neither disagree nor agree / Partially agree / Strongly agree	The original wording of the question and its response options were retained.	
	K6	A child’s oral health is related to general health.	Strongly disagree / Partially disagree / Neither disagree nor agree / Partially agree / Strongly agree	The original wording of the question and its response options were retained.	
	K7	Stains on the surfaces of teeth are the first signs of dental caries.	Strongly disagree / Partially disagree / Neither disagree nor agree / Partially agree / Strongly agree	Do you think that an opaque white or brown spot on the surface of a tooth is the first sign of dental caries?	The response options were retained
	K8	Dental caries is caused by bacteria transmitted when feeding utensils (e.g., spoons) are shared.	Strongly disagree / Partially disagree / Neither disagree nor agree / Partially agree / Strongly agree	Dental caries is an infectious disease that is transmitted by sharing feeding utensils, such as spoons.	The response options were retained
	K9	Fluoride toothpaste will help prevent dental caries in my child.	Strongly disagree / Partially disagree / Neither disagree nor agree / Partially agree / Strongly agree	The original wording of the question and its response options were retained.	
	K10	The mother’s diet during pregnancy affects the development of the baby’s teeth.	Strongly disagree / Partially disagree / Neither disagree nor agree / Partially agree / Strongly agree	The original wording of the question and its response options were retained.	
	K11	Feeding my child with breast milk will help with better growth of the face and teeth.	Strongly disagree / Partially disagree / Neither disagree nor agree / Partially agree / Strongly agree	The original wording of the question and its response options were retained.	
	K12	Habits (lip sucking, finger sucking, cloth sucking, and pacifier use) affect the development of teeth and facial bones.	Strongly disagree / Partially disagree / Neither disagree nor agree / Partially agree / Strongly agree	The original wording of the question and its response options were retained.	
	K13	About primary and permanent teeth, I think that:	Primary teeth are not important at all / Primary teeth are slightly important / Primary and permanent teeth are equally important	The original wording of the question and its response options were retained (the item was initially in the Practices dimension and was relocated to this dimension).	
Practices	P1	How often will you breastfeed your baby during the first six months of age?	Every two hours / When he/she is awake / Whenever he/she wants	During the first six months of life, how often will you breastfeed your baby?	On demand / Every two hours / Every three hours / When he/she is awake / I do not know
	P2	If you work outside the home, how will you feed the baby?	Formula milk / Breastfeeding when with the baby and formula when not with him/her / Expressed breast milk	When you work outside the home, how do you plan to feed the baby?	Expressed breast milk in a bottle / I would give formula milk / Breastfeeding when I am with the baby and formula milk when I am not with him/her / I do not know
	P3	Until what age will you feed your baby with breast milk?	Three months / Six months / Twelve months / Two years / Until the child wants	The original wording of the question was retained.	Three months / Six months / Twelve months / Two years / Until the child wants / I do not know
	P4	Who would carry out the baby’s oral care practices?	Yourself / A family member / An external caregiver / Other	The original wording of the question and its response options were retained.	
	P5	How will you feed your child up to six months of age?	Only breast milk / Mainly formula milk / Breast milk and formula milk / Other	The original wording of the question was retained.	Only breast milk / Mainly formula milk / Breast milk and formula milk / Other / I do not know
	P6	How will you feed your child from six months to one year of age?	Only breast milk / Breast milk and formula milk / Breast milk plus complementary feeding / Only complementary feeding	The original wording of the question was retained.	Only breast milk / Breast milk and formula milk / Breast milk plus complementary feeding / Only complementary feeding / I do not know
	P7	At what age will you take your child to the first dental visit?	At birth / Between six months and one year of age / After six years of age / When he/she feels pain	The original wording of the question was retained.	At birth / Between six months and one year of age / After six years of age / When he/she feels pain / I do not know
	P8	When do you think cleaning the baby’s mouth should begin?	As soon as the first tooth erupts / After the eruption of all primary teeth / The gums should be cleaned regularly after birth / I don’t know	The original wording of the question and its response options were retained.	
	P9	How often should the baby’s mouth be cleaned?	Once a day / Regularly after each feeding / Cleaning is not required before teeth erupt / I don’t know	The original wording of the question and its response options were retained.	
	P10	How will you clean your baby’s mouth before the teeth erupt?	With water / With wet gauze / With a toothbrush / With toothpaste / It should not be done	How would you clean the baby’s mouth before the teeth erupt?	With water / With a piece of wet gauze / With a toothbrush / With a silicone finger brush / With toothpaste / It should not be done /I do not know

Table 6 presents the indicators calculated after subjecting the questionnaire to expert review during the second round of evaluation. Overall, the evaluation indicators for the instrument ranged from 0.90 to 0.98, indicating excellent face and content validity. Regarding dimension-specific results, the psychosocial dimension showed scores ranging from 0.92 in the S-CVR/Ave for the clarity factor to 0.98 in the S-CVI for relevance. In the practices dimension, the indicators ranged from 0.89 in the S-CVR/Ave for clarity to 0.98 in both the S-CVI/Ave and S-CVC/Ave for relevance and essentiality (need). At the item level, all indicators exceeded the recommended thresholds for their respective indices (CVI, CVR, and CVC; see Table 2), with values ranging from 0.68 to 1.00. In conclusion, based on expert judgment, the items, dimensions, and the questionnaire as a whole demonstrate satisfactory validity.

**Table 6:** Indicators of face and content validity of the questionnaire and its components obtained in the first round of expert evaluation

Dimension	Question	Relevance			Clarity			Essentiality (Need)			Decision of the research team regarding the item, the dimension, or the instrument
		I-CVI	I-CVR	I- CVC	I-CVI	I-CVR	I- CVC	I-CVI	I-CVR	I- CVC	
Psychosocial	D1	1.00	1.00	0.98	1.00	1.00	0.97	1.00	1.00	1.00	Valid
	D2	1.00	1.00	0.98	1.00	1.00	0.98	0.96	0.92	0.99	Valid
	D3	0.88	0.76	0.87	0.84	0.68	0.87	0.84	0.68	0.92	Valid
	D4	1.00	1.00	0.97	0.96	0.92	0.96	0.96	0.92	0.99	Valid
	D5	1.00	1.00	0.97	1.00	1.00	0.97	1.00	1.00	1.00	Valid
	D6	1.00	1.00	0.98	1.00	1.00	0.98	1.00	1.00	1.00	Valid
	D7	0.96	0.92	0.95	0.96	0.92	0.94	0.92	0.84	0.95	Valid
	D8	1.00	1.00	0.94	1.00	1.00	0.94	1.00	1.00	1.00	Valid
	S-CVI/Ave										
	S-CVR/Ave										
	S-CVC/Ave	0.98	0.96	0.96	0.97	0.94	0.95	0.96	0.92	0.98	Valid
	S-CVI-UA	0.75	---	---	0.63	---	---	0.50	---	---	
Knowledge	K1	0.92	0.84	0.93	0.96	0.92	0.95	0.96	0.92	0.97	Valid
	K2	1.00	1.00	0.99	1.00	1.00	0.97	1.00	1.00	1.00	Valid
	K3	0.96	0.92	0.91	0.88	0.76	0.87	0.88	0.76	0.95	Valid
	K4	1.00	1.00	0.96	0.96	0.92	0.93	0.96	0.92	0.99	Valid
	K5	1.00	1.00	0.99	0.96	0.92	0.95	1.00	1.00	1.00	Valid
	K6	0.96	0.92	0.94	0.96	0.92	0.93	0.96	0.92	0.97	Valid
	K7	1.00	1.00	0.98	0.88	0.76	0.92	0.96	0.92	0.99	Valid
	K8	0.92	0.84	0.93	0.84	0.68	0.85	0.92	0.84	0.96	Valid
	K9	1.00	1.00	0.98	0.96	0.92	0.94	1.00	1.00	1.00	Valid
	K10	1.00	1.00	0.96	1,00	1,00	0.96	0.96	0.92	0.99	Valid
	K11	1.00	1.00	0.98	0.96	0.92	0.92	0.96	0.92	0.99	Valid
	K12	0.96	0.92	0.98	0.92	0.84	0.93	0.96	0.92	0.97	Valid
	K13	1.00	1.00	0.97	1.00	1.00	0.97	1.00	1.00	1.00	Valid
	S-CVI/Ave										
	S-CVR/Ave										
	S-CVC/Ave	0.98	0.96	0.96	0.94	0.89	0.93	0.96	0.93	0.98	Valid
	S-CVI-UA	0.62	---	---	0.23	---	---	0.23	---	---	
Practices	P1	0.96	0.92	0.93	0.92	0.84	0.92	1.00	1.00	1.00	Valid
	P2	0.96	0.92	0.96	0.92	0.84	0.93	0.96	0.92	0.99	Valid
	P3	0.92	0.84	0.94	0.92	0.84	0.96	0.92	0.84	0.96	Valid
	P4	0.92	0.84	0.93	0.92	0.84	0.93	0.96	0.92	0.99	Valid
	P5	0.92	0.84	0.93	0.96	0.92	0.97	0.96	0.92	0.99	Valid
	P6	0.96	0.92	0.95	0.96	0.92	0.94	0.96	0.92	0.99	Valid
	P7	0.96	0.92	0.96	0.96	0.92	0.96	1.00	1.00	1.00	Valid
	P8	1.00	1.00	0.97	1.00	1.00	0.94	1.00	1.00	1.00	Valid
	P9	0.92	0.84	0.95	0.96	0.92	0.96	0.96	0.92	0.99	Valid
	P10	0.96	0.92	0.95	1.00	1.00	0.97	1.00	1.00	1.00	Valid
	S-CVI/Ave										
	S-CVR/Ave										
	S-CVC/Ave	0.95	0.90	0.95	0.95	0.90	0.95	0.97	0.94	0.99	Valid
	S-CVI-UA	0.10	---	---	0.20	---	---		---	---	
S-CVI/Ave (Overall)											
S-CVR/Ave (Overall)											
T- CVC (Overall)	0.97	0.94	0.96	0.95	0.91	0.94	0.97	0.93	0.98	Valid	
S-CVI-UA (Overall)	0.48	----	----		0.32		----	----	0.32	----	----	

However, some experts once again made additional suggestions, which were reviewed by the research team and subjected to editorial refinement. This process led to the development of the final version of the instrument, available for researchers (Supplementary Table).

## DISCUSSION

This study facilitated the design of a Spanish-language questionnaire to assess the knowledge and practices of pregnant women and mothers regarding their children’s oral health. This instrument is not only useful for conducting research in specific social and geographical contexts-thereby enhancing the understanding of local realities-but also serves as a vital resource for developing strategies to promote oral health within the mother-child dyad.

Additional elements included in this study encompass the psychosocial dimension, which involve attitudes and opinions toward oral hygiene and health, as well as feelings related to the importance and satisfaction of breastfeeding and feeding practices during early childhood. The scientific literature highlights the role of maternal feeding-particularly breastfeeding-in promoting craniofacial development, protecting against malocclusions ^51^, and reducing the risk of dental caries among children when integrated with adequate oral hygiene and health practices. ^52^ Furthermore, the questions across various dimensions consider the bond established within the mother-child dyad. This bond is crucial for oral health care, as the preventive and educational behaviors transmitted by the mother directly impact the child’s oral well-being. ^18^ Strengthening this bond fosters the adoption of healthy habits from an early age, leading to reduced risks and improved oral health outcomes.

Beliefs, along with KAP related to oral health, are subjects of debate and inquiry among pregnant women, mothers, and other caregivers involved in child-rearing. To the best of our knowledge, this is one of the few questionnaires developed in Colombia and, more broadly, in Latin America, specifically designed to assess the knowledge of mothers and/or pregnant women regarding their children’s oral health.

Supporting this assertion, a review of studies published in indexed journals in this region over the past decade reveals research with certain conceptual or methodological limitations that could be improved, thereby justifying the design of the present instrument. For instance, many studies focus on pregnant women’s knowledge and practices regarding their own oral health and hygiene ^53^^,^^54^, while others are centered on children older than three years ^55^^,^^56^, or do not specify the child population reference. ^57^ Additionally, the data collection instruments and their processes of content and face validity are often not clearly described. ^54^^,^^58^ Notably, the studies referenced for the design and validation of the questionnaire proposed in this research were conducted in countries such as Qatar and Saudi Arabia. ^27^^,^^28^^,^^37^^,^^39^ Therefore, the design of questionnaires based on the KAP model must consider the specific characteristics of the target populations, and adopting a cultural perspective allows for a better understanding of local needs and realities.

It is important to outline the strengths and limitations of this study to delineate its conceptual and methodological scope. The development of this questionnaire incorporated a variety of tools and techniques for collecting both quantitative and qualitative data, enabling the triangulation of items based on multiple primary and secondary information sources. Experts from other Latin American countries, such as Peru and Ecuador, were also consulted, which supports the cultural adaptation of the questionnaire for use in other Spanish-speaking countries. Regarding the study’s limitations, it should be noted that the construction and design of the instrument involved an initial phase of validation through expert consensus, complemented by additional analytical procedures. However, the assessment of the instrument’s psychometric properties (reliability and validity) in a larger sample with the necessary statistical power-consistent with recommendations in the scientific literature-remains pending.

Future research should incorporate qualitative and mixed methods approaches to address complex issues in oral health, providing a comprehensive and integrative perspective. Additionally, intervention studies should evaluate the impact of educational strategies and health promotion and prevention programs on the social and health realities of various social groups.

## CONCLUSION

By employing diverse quantitative and qualitative methodologies and consulting both academic experts and the study population, we successfully designed and validated a questionnaire assessing mothers’ knowledge and practices regarding oral hygiene and oral health in children under two years of age. This instrument incorporates a cultural perspective, emphasizes the mother-child bond, and addresses key aspects related to maternal feeding and breastfeeding during early life. The questionnaire is available for research use and can be adapted to various social contexts across Latin America. Overall, it represents a methodological contribution to maternal and child oral health research by providing a culturally sensitive and validated tool with potential applicability in both research and health promotion programs.

## SUPPLEMENTARY TABLE.FINAL QUESTIONNAIRE (IN SPANISH)

**Table:**

Encabezado de la encuesta (puede ser modificado a conveniencia de los objetivos del estudio) Querida participante: La invitamos a responder esta breve encuesta que se desarrolla en el marco de los conocimientos para el cuidado de su futuro hijo. Las preguntas tienen una única respuesta y pretenden conocer su percepción sobre los diferentes aspectos.							
Datos sociodemográficos							
Estas variables pueden ser adaptadas al contexto de la investigación, pero son importantes para caracterizar el contexto social del estudio.							
Edad (años cumplidos)		Estado civil	Nivel educativo máximo alcanzado			Número de hijos	Ocupación
Estrato socioeconómico		País de origen	Lugar de residencia			Grupo étnico	
Dimensión psicosocial							
Esta dimensión se concentra en preguntas o afirmaciones relacionadas con actitudes, y en el vínculo entre la madre y el bebé teniendo en cuenta procesos relacionados con higiene oral, lactancia materna y salud oral en niños menores de dos años. En los siguientes enunciados, encontrará afirmaciones cuyo interés es conocer su punto de vista. Por favor, marque con una X. Elija solo una respuesta.							
D1. La lactancia materna aumenta la unión madre-hijo.	Estoy totalmente en desacuerdo	Estoy parcialmente en desacuerdo		Ni en desacuerdo ni en acuerdo	Estoy parcialmente de acuerdo		Estoy totalmente de acuerdo
D2. Mantener la salud oral de un hijo es un deber de los padres.	Estoy totalmente en desacuerdo	Estoy parcialmente en desacuerdo		Ni en desacuerdo ni en acuerdo	Estoy parcialmente de acuerdo		Estoy totalmente de acuerdo
D3. Si fuera necesario dar biberón (tetero), ¿cómo se sentiría?			Totalmente insatisfecha	Insatisfecha	Neutral	Satisfecha	Totalmente satisfecha
D4. Alimentar con pecho a su bebé (amamantar), la hace sentir:			Totalmente insatisfecha	Insatisfecha	Neutral	Satisfecha	Totalmente satisfecha
D5. Adquirir conocimientos en prácticas de cuidado e higiene oral para su bebé es:			Para nada útil	No muy útil	Neutral	Algo útil	Muy útil
D6. Aprender sobre cómo alimentar a su bebé durante el primer año de vida es:			Para nada útil	No muy útil	Neutral	Algo útil	Muy útil
D7. Considera que amamantar (lactar) a su bebé:			No es importante	Es poco importante	Neutral	Es importante	Es muy importante
D8. ¿Considera difícil continuar con la lactancia materna cuando trabaja fuera de casa?			Estoy totalmente en desacuerdo	Estoy parcialmente en desacuerdo	Ni en desacuerdo ni en acuerdo	Estoy parcialmente de acuerdo	Estoy totalmente de acuerdo
Dimensión conocimientos Esta dimensión se concentra en serie de afirmaciones o preguntas que evalúan el conocimiento materno sobre alimentación, higiene y salud bucal durante el primer año de vida. En los siguientes enunciados, encontrará afirmaciones cuyo interés es conocer su punto de vista. Por favor, marque con una X. Elija solo una respuesta.							
K1. ¿Piensa que las leches de formula (leche de tarro) alimentan igual que la leche materna?			Estoy totalmente en desacuerdo	Estoy parcialmente en desacuerdo	Ni en desacuerdo ni en acuerdo	Estoy parcialmente de acuerdo	Estoy totalmente de acuerdo
K2. La leche materna tiene mayores beneficios que la alimentación con leche de fórmula			Estoy totalmente en desacuerdo	Estoy parcialmente en desacuerdo	Ni en desacuerdo ni en acuerdo	Estoy parcialmente de acuerdo	Estoy totalmente de acuerdo
K3. Las madres aprenden de forma natural y por instinto materno cómo amamantar al bebé:			Estoy totalmente en desacuerdo	Estoy parcialmente en desacuerdo	Ni en desacuerdo ni en acuerdo	Estoy parcialmente de acuerdo	Estoy totalmente de acuerdo
K4. Los bebés que se alimentan con leche materna son más sanos que aquellos que se alimentan con leche de fórmula.			Estoy totalmente en desacuerdo	Estoy parcialmente en desacuerdo	Ni en desacuerdo ni en acuerdo	Estoy parcialmente de acuerdo	Estoy totalmente de acuerdo
K5. Alimentar en la noche a su hijo con tetero de leche de fórmula o bebida que contenga azúcar afecta sus dientes.			Estoy totalmente en desacuerdo	Estoy parcialmente en desacuerdo	Ni en desacuerdo ni en acuerdo	Estoy parcialmente de acuerdo	Estoy totalmente de acuerdo
K6. La salud oral del niño se relaciona con la salud general.			Estoy totalmente en desacuerdo	Estoy parcialmente en desacuerdo	Ni en desacuerdo ni en acuerdo	Estoy parcialmente de acuerdo	Estoy totalmente de acuerdo
K7. ¿Considera que al observar una mancha blanca o café opaca en la superficie de un diente es el primer signo de caries dental?			Estoy totalmente en desacuerdo	Estoy parcialmente en desacuerdo	Ni en desacuerdo ni en acuerdo	Estoy parcialmente de acuerdo	Estoy totalmente de acuerdo
K8. La caries es una enfermedad que se trasmite al compartir utensilios de alimentación, por ejemplo, cucharas.			Estoy totalmente en desacuerdo	Estoy parcialmente en desacuerdo	Ni en desacuerdo ni en acuerdo	Estoy parcialmente de acuerdo	Estoy totalmente de acuerdo
K9. La crema dental con flúor, ayudará a prevenir la caries dental en su hijo.			Estoy totalmente en desacuerdo	Estoy parcialmente en desacuerdo	Ni en desacuerdo ni en acuerdo	Estoy parcialmente de acuerdo	Estoy totalmente de acuerdo
K10. La alimentación de la madre durante el embarazo afecta el desarrollo de los dientes del bebé.			Estoy totalmente en desacuerdo	Estoy parcialmente en desacuerdo	Ni en desacuerdo ni en acuerdo	Estoy parcialmente de acuerdo	Estoy totalmente de acuerdo
K11. Alimentar a su hijo con lactancia materna ayudará a un mejor crecimiento de la cara y sus dientes			Estoy totalmente en desacuerdo	Estoy parcialmente en desacuerdo	Ni en desacuerdo ni en acuerdo	Estoy parcialmente de acuerdo	Estoy totalmente de acuerdo
K12. Los hábitos (succión de labio, dedo, tela y uso de chupos) afectarán el desarrollo de los dientes y los huesos de la cara			Estoy totalmente en desacuerdo	Estoy parcialmente en desacuerdo	Ni en desacuerdo ni en acuerdo	Estoy parcialmente de acuerdo	Estoy totalmente de acuerdo
K13. Sobre los dientes de leche y permanentes, piensa que:			Los dientes de leche no son nada importantes	Los dientes de leche son ligeramente importantes.			Dientes de leche y permanentes son igual de importantes
Dimensión prácticas Esta dimensión se concentra en preguntas o afirmaciones que evalúan las diferentes prácticas que realizan las madres para mantener o mejorar la salud oral y la higiene oral de sus hijos. En los siguientes enunciados, encontrará afirmaciones cuyo interés es conocer su punto de vista. Por favor, marque con una X. Elija solo una respuesta.							
P1. En los primeros seis meses de vida del bebé, usted lo amamantará:			A libre demanda	Cada dos horas	Cada tres horas	Cuando esté despierto	No lo sé
P2. Cuando trabaje fuera de casa, ¿cómo piensa alimentar al bebé?			Leche materna extraída en tetero (biberón)	Le daría leche de tarro en tetero/biberón (leche de fórmula)	Lactancia materna cuando estoy con el bebé y leche de tarro cuando no estoy con él		No lo sé
P3. ¿Hasta qué edad alimentará a su bebé con leche materna?		Tres meses	Seis meses	Doce meses	Dos años	Hasta que el niño quiera	No lo sé
P4. ¿Quién realizaría las prácticas de cuidado oral del bebé?			Usted misma	Un familiar	Cuidador externo	Otro	
P5. ¿Cómo va a alimentar a su hijo hasta los seis meses?			Solo con leche materna	Con leche de fórmula principalmente	Lactancia materna y leche de fórmula	Otro	No lo sé
P6. ¿Cómo alimentará a su hijo, de los seis meses al año?			Solo con leche materna	Lactancia materna y leche de fórmula	Lactancia materna más alimentación complementaria	Solo alimentación complementaria	No lo sé
P7. ¿A qué edad va a llevar a su hijo a la primera visita al odontólogo?			Al nacer	Entre los seis meses y el año	Después de los seis años	Cuando sienta algún dolor	No lo sé
P8. ¿Cuándo piensa que se debería iniciar la limpieza de la boca del bebé?			Tan pronto como le sale el primer diente	Después de la erupción de todos los dientes primarios	Las encías deben limpiarse regularmente después del nacimiento	No lo sé	
P9. ¿Cada cuanto debería limpiar la boca del bebé?			Una vez al día	Regularmente después de cada alimentación	No se requiere limpieza antes de que salgan los dientes	No lo sé	
P10. ¿Con qué elemento realizaría la limpieza de la boca del bebé antes que le salgan los dientes?	Con agua	Con gasa mojada	Con cepillo de dientes	Con dedal para bebés	Con crema dental	No debería	No lo sé
